# The Precipitation Behavior of a Cu-Ni-Si Alloy with Cr Addition Prepared by Heating-Cooling Combined Mold (HCCM) Continuous Casting

**DOI:** 10.3390/ma15134521

**Published:** 2022-06-27

**Authors:** Xianghao Meng, Guoliang Xie, Wenli Xue, Yilei Fu, Rui Wang, Xinhua Liu

**Affiliations:** 1 Key Laboratory for Advanced Materials Processing (MOE), Institute of Advanced Materials and Technology, University of Science and Technology Beijing, Beijing 100083, China; g20199382@xs.ustb.edu.cn (X.M.); s20191347@xs.ustb.edu.cn (Y.F.); d202110678@xs.ustb.edu.cn (R.W.); 2 Beijing Laboratory of Metallic Materials and Processing for Modern Transportation, Institute for Advanced Materials and Technology, University of Science and Technology Beijing, Beijing 100083, China; 3 Beijing Advanced Innovation Center for Materials Genome Engineering, Institute for Advanced Materials and Technology, University of Science and Technology Beijing, Beijing 100083, China; 4 State Key Laboratory for Advanced Metals and Materials, University of Science and Technology Beijing, Beijing 100083, China; g20199201@xs.ustb.edu.cn

**Keywords:** discontinuous precipitation, nucleation, aging treatment, precipitation

## Abstract

A Cu-Ni-Si alloy containing (Ni + Si) ≥ 5 wt.%, with the addition of Cr, is fabricated by HCCM continuous casting and two steps of aging treatment. The evolution of the microstructures and precipitations, as well as the effect of Cr atoms, is studied in this paper. An excellent combination of mechanical property (hardness HV 250–270) and electrical conductivity (46–47 %IACS) is obtained by the first step aging at 500 °C for 0.25 h and the second step aging at 450 °C for 1 h. The cold rolling and aging process are directly conducted on the solution treated specimens fabricated by HCCM continuous casting process without hot deformation, since the excellent homogeneity of matrix is obtained by solution treatment with δ-Ni_2_Si precipitates dissolved. It is found that the formation of discontinuous precipitation is suppressed by the formation of Cr_3_Si cores of 5–10 nm before the formation δ-Ni_2_Si. Then, the nucleation and growth of δ-Ni_2_Si precipitates occurs around the boundaries of these Cr_3_Si cores, leading to an enhanced nucleation rate. This study provides a promising direction for the design and optimization of Cu-Ni-Si alloys based on the further understanding of the effect of the addition of Cr.

## 1. Introduction

The precipitation hardening Cu-Ni-Si alloys are widely used for electrical applications such as lead frames, contactors and electrical connectors, with requirement of a combination of good electrical and mechanical properties [1,2,3]. The precipitation process of Cu-Ni-Si alloy is started and controlled by spinodal decomposition process (i.e., the formation of G.P. zone containing solute atoms enriched and depleted regions due to uphill-diffusion) [4]. There are mainly two kinds of precipitates in the Cu-Ni-Si alloys (i.e., β-Ni_3_Si and δ-Ni_2_Si) [5,6]. It has been reported [7] that the precipitation sequence of G.P. zones, β-Ni_3_Si with DO_22_ (or L1_2_) ordered precipitates, δ-Ni_2_Si with orthogonal structure occurs is observed during the aging process of Cu-Ni-Si alloy. The strength is enhanced mainly due to the formation of nano-meter precipitates of δ-Ni_2_Si. The strengthening effect of these semi-coherent precipitates is mainly attributed to the dislocation by passing mechanism, which is also called Orowan mechanism [8]. Therefore, the strengthening effect is determined by the amount, size and distribution of the precipitates. The strength will be decreased gradually after the peak strength of aging process is reached, due to the growth of the precipitates of δ-Ni_2_Si. The electrical conductivity of the alloy is enhanced as the precipitation process continues, due to the decrease of the solute atoms in matrix.

The combination of higher strength and better electrical conductivity is always pursued in Cu-Ni-Si alloys for the advanced industrial applications. Many attempts have been tried for the further improvement in the electrical and mechanical properties of Cu-Ni-Si alloys by the addition of the third trace elements such as Cr, Co, Mg, Al and P to Cu-Ni-Si alloys [9,10,11,12,13]. Thereafter, the effects of these alloying elements on the properties and structure of Cu-Ni-Si alloys have been studied in many previous works [14,15,16]. Trace amount of P can form the Ni_3_P precipitates and suppress the discontinuous precipitation reaction, resulting in enhancement of both strength and elongation [17]. The addition of Mg and Al can improve the strength by decreasing the inter-precipitate spacing, and enhance the stress-relaxation resistance due to the Mg-atom-drag effect on dislocations [18,19]. The addition of Co, as reported by several previous works [20,21], can partially replace the Ni atoms in δ-Ni_2_Si precipitates to form δ-(Ni, Co)_2_Si, suppress spinodal decomposition process, and promote the precipitation of precipitates, leading to the improvement of the mechanical properties of the material. Among these alloying elements, Cr is considered as an effective alloying element for the enhancement in the strength of Cu matrix without impairing the electrical conductivity, which is attributed to the very low solubility of Cr in Cu matrix [22]. Meanwhile, the addition of Cr is expected to remove excessive Si in the Cu matrix due to the formation of Cr_3_Si phase during solidification and aging treatment [23,24], which can promote the precipitation of Si and Ni [13,25]. Since Cr atoms are present principally in the form of particles with several micro-meters in size, it affects primarily the temperature stability of properties, particularly at temperatures higher than that at which the Ni_2_Si phase is dissolved. The Cr_3_Si precipitates can improve the high temperature stability of properties by impeding the grain growth during heat treatment [26,27], due to the stable structure at elevated temperatures. The addition of Cr atoms can also improve the plasticity and electrical conductivity, when nickel and silicon are not present in the appropriate stoichiometric proportions to form Ni_2_Si. However, the effect of Cr atoms on the formation and growth of precipitation is still unclear, especially on the precipitation process concerning continuous precipitates and discontinuous precipitates.

On the other hand, a number of studies [28,29] have been conducted to improve the strength of Cu-Ni-Si alloys by increasing the contents of Ni (up to 6–8 wt.%) and Si (1–2 wt.%). Whereas, the conductivity of the alloy will be inevitably reduced since more solute atoms are left in matrix as the contents of Ni and Si increase [30]. In addition, the increase of (Ni + Si) content will promote the generation of discontinuous precipitates, which is harmful to the mechanical properties of alloy [31]. Therefore, it is important to study the effect of elements such as Cr on the precipitation process, for the optimization and design of Cu-Ni-Si alloys.

In this paper, the inhibition on discontinuous precipitation by the addition of Cr on the Cu-Ni-Si alloy is studied, in order to obtain the optimization of precipitation process and the improvement of properties. The heating-cooling combined mold (HCCM) method of the alloy is conducted for the fabrication of the Cu-Ni-Si alloy in order to obtain a supersaturated solid solution matrix containing more uniformly distributed alloying elements, which offers a basis for the simplification of the fabrication process. The evolution of the microstructures and precipitations, as well as the corresponding variation of mechanical properties and electrical conductivities are studied herein.

## 2. Experimental Procedure

### 2.1. The Fabrication and Aging Treatment of Materials

**Figure 1:**
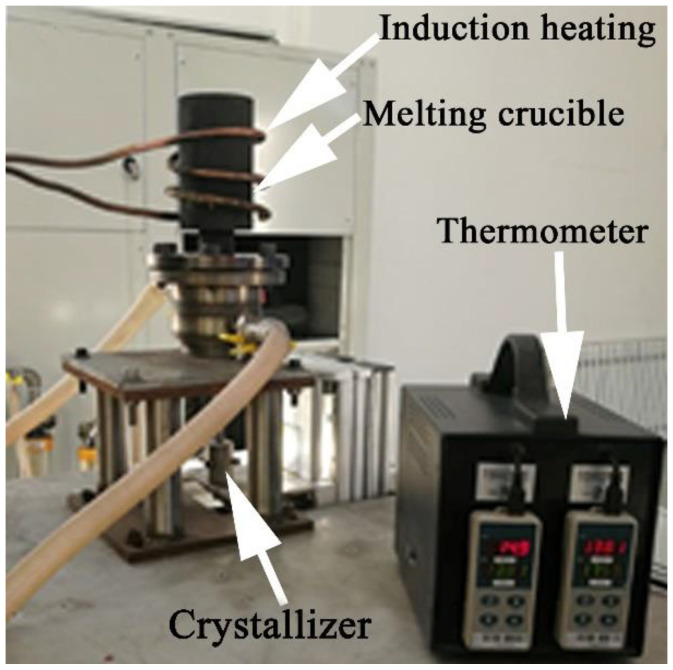
The illustration of HCCM continuous casting device.

The ingot of a Cu-Ni-Si alloy containing 4.74 wt.% Ni, 1.19 wt.% Si, 0.096 wt.% Cr and the balance of Cu is prepared by HCCM continuous casting method [32] with melt temperature of 1300 °C, cooling water flow rate 600 L/h and casting speed 1 mm/s, as illustrated in Figure 1. The ingot is then solution treated at 1000 °C for 1 h to obtain a homogenized supersaturated solid solution. The ingot is directly deformed into plates by cold rolling of 90% reduction. A two-step aging process is conducted herein for the further improvement in the precipitation process (i.e., first step of aging treatment at 500 °C, followed by 70% cold rolling and second step of aging treatment for different time at 450 °C).

### 2.2. Microstructure Observation

Samples for microstructural and mechanical property examinations are cut from the sheets subjected to different aging treatments, parallel to the rolling direction. Scanning electron microscopy (SEM, JEOL-7900 field emission scanning electron microscope, JEOL, Tokyo, Japan), Transmission electron microscopy (TEM, Tecnai G^2^ F20 transmission electron microscope, FEI, Hillsboro, FL, USA) are employed to investigate the microstructural evolution. The TEM foils for TEM observation are prepared according to standard electro-polishing techniques for Cu alloys.

### 2.3. Mechanical Properties

The tensile tests are carried out using a CMT4105 electronic universal material tester (MTS, USA) under a strain rate of 2 mm/min to obtain yield strength and tensile strength. Samples with dimensions of 15 mm × 15 mm in size are cut from the sheets for the measurement of electrical conductivity and hardness. The electrical conductivity of each aged specimen is determined by a Sigma 2008-A digital eddy current conductivity meter (HFD, Beijing, China) to investigate the precipitation inetics. The Vickers hardness tests are carried out using a HXD-1000TM/LCD Vicker’s hardness tester (Shanghai optical instrument factory, Shanghai, China) under the load of 300 g with holding time of 15 s. The measurement of the electrical conductivity and hardness is repeated at least five times to minimize the errors in the experimental data.

## 3. Results

### 3.1. The Variation of the Mechanical Properties and Electrical Conductivities

**Figure 2:**
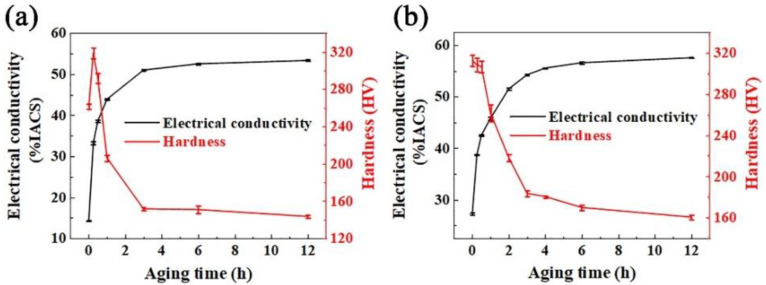
The variation of hardness and electrical conductivity with increasing aging time, (**a**) the first step (500 °C) and (**b**) the second step (450 °C) of aging treatment.

Figure 2 demonstrates the variation of hardness and electrical conductivity of the Cu-Ni-Si alloy during the first step of aging treatment at 500 °C (in Figure 2a) and the second step of aging treatment at 450 °C (in Figure 2b) plotted against the aging time. The age-hardening response during the first step of aging process of the alloy can be roughly divided into three stages according to Figure 2a (i.e., the rapid increase of hardness, the peak strengthening hardness (~0.25 h), and the decrease of hardness with aging time larger than 0.25 h). The hardness is found deceasing rapidly with the aging time in range of 0.5–3 h, probably due to the softening effect of the matrix and the coarsening of the precipitates. It is also suggested by the rapid increase of hardness in the earlier aging stage that, the nucleation of precipitation is greatly promoted by the cold rolling process.

The variation of electrical conductivity is believed to be proportional to the contents of precipitation in Cu-Ni-Si alloys [29,33,34,35]. The experimental data of micro-hardness and electrical conductivity are plotted in Figure 2, containing the average value and error bars obtained by the measurements that have been repeated five times. It is found that the content of precipitation is increased rapidly with the aging time increasing from 0 to 3 h, as revealed by the variation of electrical conductivity in Figure 2a. The highest precipitation rate is also obtained with aging time of 0.25 h. So the first step of aging treatment of 500 °C for 0.25 h is chosen with the following second step of aging, in order to optimize the precipitation rate. The variation of hardness and electrical conductivity with increasing aging time during the second step of aging process at 450 °C is shown in Figure 2b. It is found by the variation of hardness that, the hardness is reduced quite slowly with aging time increasing from 0 to 0.5 h, suggesting the coexistence of nucleation of precipitates and softening of matrix. Then the hardness is reduced rapidly when the aging time is continuously increased, due to the coarsening of precipitates and the decreasing of nucleation rate. The electrical conductivity is found increasing rapidly with the aging time increasing from 0 to 2 h, suggesting very rapid precipitation formation rate. Therefore, the optimization of the properties can be realized the two steps aging treatment since the precipitation rate is enhanced, leading to the improvement of the size and distribution of precipitates. Excellent combination of mechanical property (hardness HV 250–270) and electrical conductivity (46–47% IACS) is obtained by the first step aging at 500 °C for 0.25 h and the second step aging at 450 °C for 1 h, as compared to several previous works [6,7,8,9]. The yield strength of 835 MPa, tensile strength of 845 MPa with elongation of ~5% is obtained by tensile tests, which is in good accordance with the data of hardness.

### 3.2. The Evolution of Microstructures

**Figure 3:**
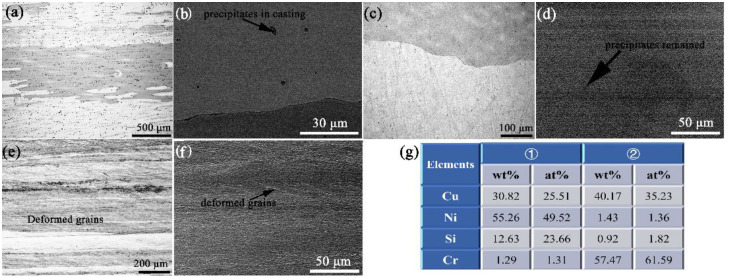
The evolution of microstructures of the Cu-Ni-Si alloy during the fabrication process, containing SEM photographs of (**a**) HCCM casting, (**b**) the magnified casting microstructure, (**c**) solution treated, (**d**) the magnified solution treated microstructure, (**e**) cold rolling, (**f**) magnified cold rolling microstructure, and (**g**) the EDS data of the position marked in (**a**,**b**).

The evolution of microstructures of this Cu-Ni-Si alloy during the fabrication process is shown in Figure 3, containing the SEM photographs of specimens with different states, i.e., HCCM casting Figure 3a,b, solution treated Figure 3c,d, and cold rolling Figure 3e,f. Columnar grains are formed and observed in the HCCM casting specimens, containing some small δ-Ni_2_Si precipitates, as seen in Figure 3b. The Ni/Si atomic ratio is ~2.05, containing small amount of Cr, as shown by the EDS results in Figure 3g. These δ-Ni_2_Si precipitates are formed during the solidification and cooling process of the ingot, with size about 5–15 μm. Both the size and amount of these δ-Ni_2_Si precipitates are found smaller than those in the traditional casting ingots, due to the quite rapid cooling rate of the HCCM continuous casting method. Therefore, most of the δ-Ni_2_Si precipitates are dissolved back into matrix during the solution treatment, resulting in a homogenized matrix only containing several small Cr enriched particles with size of 1–2 μm, as shown in the EDS results in Figure 3g. The ingot is directly cold rolled into plates, without hot deformation. The microstructure of the cold rolling specimens is revealed in Figure 3e,f, containing the deformed grains along the rolling direction.

**Figure 4:**
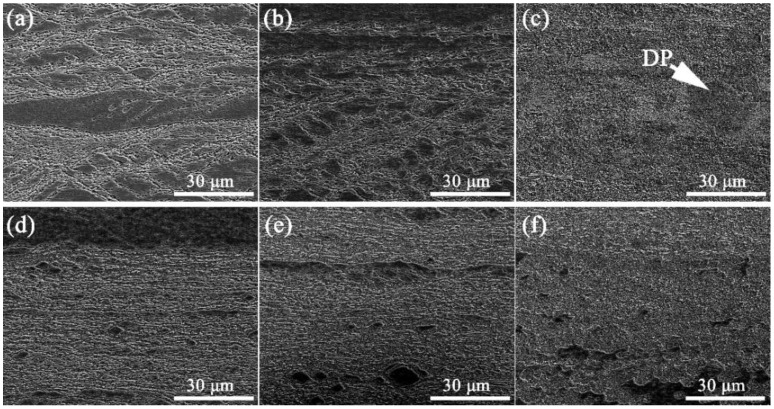
The evolution of microstructures of the Cu-Ni-Si alloy during the two steps of aging treatment containing the first step aging at 500 °C for (**a**) 0.25 h, (**b**) 0.5 h, and (**c**) 3 h, and the second step aging at 450 °C for (**d**) 0.25 h, (**e**) 0.5 h, and (**f**) 3 h.

The evolution of microstructure during the aging process is revealed in Figure 4. It is observed in Figure 4a–c that, the discontinuous precipitation is apparently inhibited. It has been reported elsewhere [36,37] that discontinuous precipitation (DP) shall occur at 500 °C when (Ni + Si) content is higher than 5 wt.%. Different to the Cu-Ni-Si alloy containing the same chemical compositions but without Cr, no DP microstructure is observed in the early stage of aging process, as seen in Figure 4a,b. Typical classic DP microstructure is observed in specimen corresponding to the first aging step at 500 °C for 3 h, as revealed in Figure 4c. The amount of DP areas, as pointed by the white arrow, is found much less than that has been reported elsewhere for the Cu-Ni-Si alloys without Cr, suggesting the distinct inhibition on the DP process of Cr atoms.

The microstructure evolution of the specimen during the second step of aging treatment at 450 °C is revealed in Figure 4d–f. It can be seen that the recrystallization occurs during the second step of aging process, resulting in the softening behavior as shown in Figure 2b.

### 3.3. The Precipitation Process

**Figure 5:**
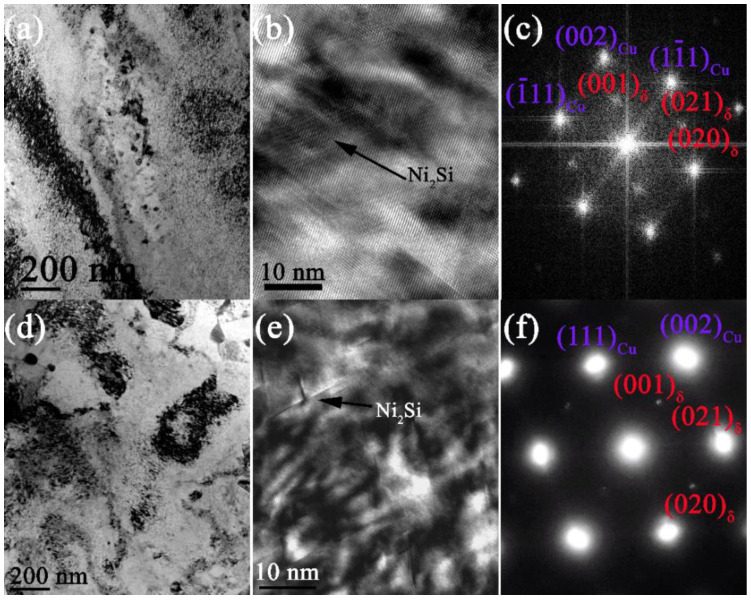
The evolution of precipitation of the Cu-Ni-Si alloy during the two steps of aging treatment containing the first step aging at 500 °C for (**a**) 0.25 h, (**b**) the magnified photograph with (**c**)selected areas diffraction patterns (SADP), and (**d**) 0.5 h, (**e**) the magnified photograph with (**f**) SADP.

**Figure 6:**
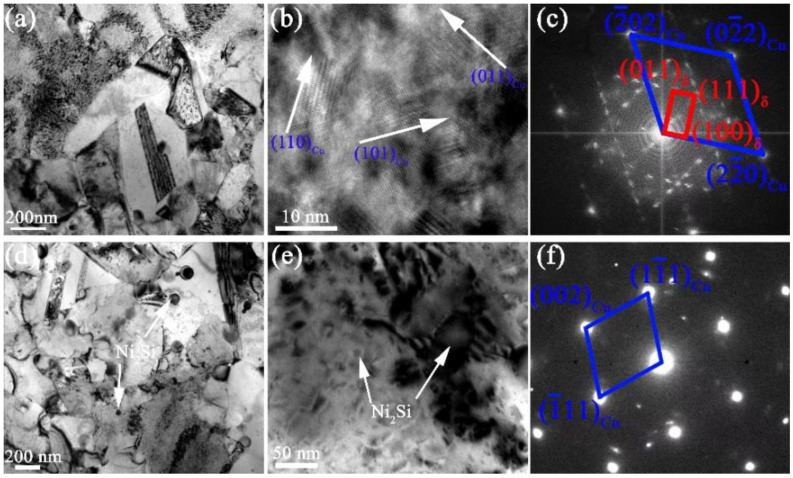
The evolution of precipitation of the Cu-Ni-Si alloy during the second step of aging at 450 °C for (**a**) 2.0 h and (**b**) the magnified photograph with (**c**) SADP and (**d**) 12.0 h, and (**e**) the magnified photograph with (**f**) SADP.

It is suggested by the evolution of microstructures in Figure 4 that, the precipitation of this alloy in the two steps of aging process mainly contains the continuous precipitates (CP), i.e., δ-Ni_2_Si. The precipitates that are formed in the first and second step of aging process are revealed by the TEM photographs in Figure 5 and Figure 6. Refined precipitates of δ-Ni_2_Si are found in Figure 5a,b, with small size of ~5–10 nm. The orientation relationship (OR) of these precipitates and matrix is revealed by the selected areas diffraction patterns (SADP): [110]_Cu_//[100]δ and (001)_Cu_//(001)_δ_, according to the SADP in Figure 5c. Therefore, the precipitates are believed to be disc-shaped, since the growth in the [001]δ direction is more restricted than the [100]δ and [010]δ directions due to the difference of misfit along these directions. The misfit along [100]δ and [010]δ directions are calculated to be close to each other, i.e., ~2.41% and ~2.81%, which are much smaller than that along [001]δ of 8.26%, according to the reference [34]. The size of these δ-Ni_2_Si precipitates is found remaining almost the same when the aging time is increased to 0.5 h, as seen in Figure 5d,e. The same OR is found in Figure 5f, suggesting no transformation of these precipitates. It is believed that the nucleation rate is fairly high and the growth is slow in the early stage of the first step of aging process, by comparing the observation in Figure 5a–d. Therefore, the decrease of hardness when aging time is increased to 0.5 h is mainly due to the crystallization of matrix. The formation of precipitation during the second step of aging process is revealed by the TEM photographs in Figure 5e,f.

It is found that many refined δ-Ni_2_Si precipitates are formed in the early stage of aging process, as shown in Figure 6a,b, with the size of only about 10 nm. It shall be noticed that, a OR between precipitates and matrix of (110)_Cu_//(100)_δ_, [111]_Cu_//[011]_δ_. It can also be seen in Figure 6b that, precipitates along the three crystal planes of <110>, i.e., [110], [101] and [011] planes of Cu matrix, are observed in the alloy as marked by the white arrow in Figure 6b, which is in good accordance with the SADP in Figure 6c. The size of these precipitates is found increasing with the prolonging aging time. For instance, the size of δ-Ni_2_Si precipitates are increased to more than 1 μm (as seen in Figure 6d), resulting in the loss of OR between precipitates and matrix, as shown in Figure 6e,f.

## 4. Discussion

### 4.1. The Influence of HCCM on Deformation and Solution Process

It can be seen from the experimental results above that the Cu-Ni-Si alloy containing Cr element is fabricated by the process of HCCM continuous casting, solution treatment, cold rolling and two steps of aging process. The DP is inhibited by the addition of Cr, according to the microstructure evolution. The precipitation process is similar to the other Cu-Ni-Si alloys reported elsewhere [5].

**Figure 7:**
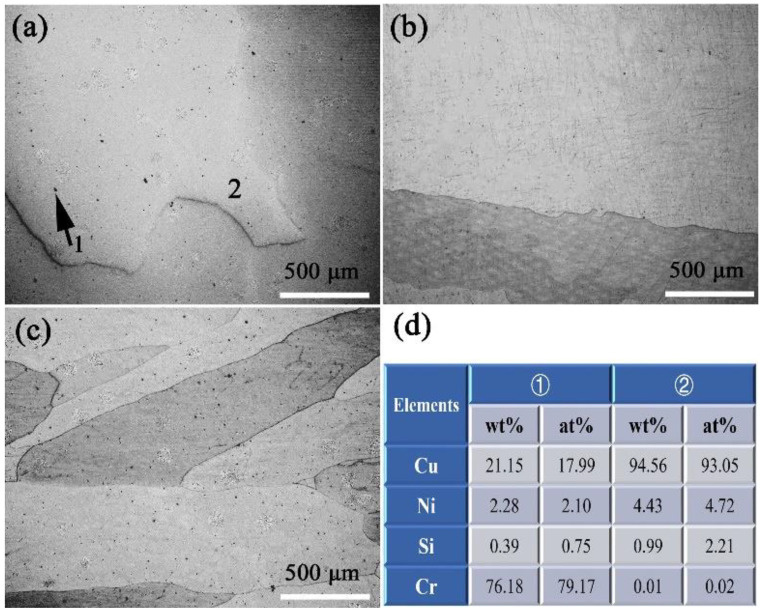
The evolution of microstructures of the Cu-Ni-Si alloy with increasing temperatures of solution treatment, containing SEM photographs at (**a**) 980 °C, (**b**) 1000 °C, and (**c**) 1020 °C, with the corresponding EDS data. (**d**) the EDS of position 1 and 2 in (**a**).

**Figure 8:**
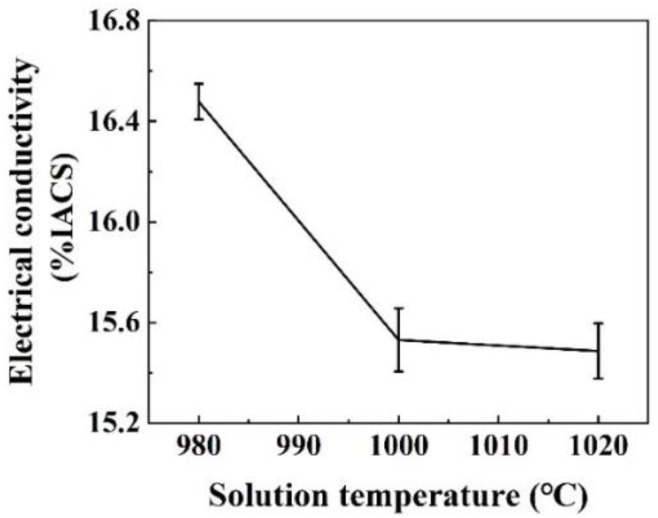
The variation of electrical conductivity with the increasing solution treatment temperature.

As mentioned above, the δ-Ni_2_Si precipitates that are formed in the solidification and cooling process of the casting specimens are much smaller than those reported in traditional casting methods. The chemical composition obtained by HCCM continuous casting are also more uniformly distributed than the traditional casting, which can be proved by the microstructure in Figure 3. Therefore, the solution treatment is simplified to obtain a homogenized matrix. The SEM photographs of solution treated at different temperatures are compared in Figure 7a–c. All of the δ-Ni_2_Si precipitates are dissolved back into the matrix for the solution treatment at 980–1020 °C, as seen in Figure 7. However, some Cr enriched phases containing ~76.18 wt.% Cr as shown by Figure 7d are found left in the specimen solution treated at 980 °C, but will be dissolved when solution temperature is increased to 1000–1020 °C, according to the comparison of Figure 7a–c. Then it is believed that the specimen can be homogenized by solution treatment at 1000 °C for only 1 h, which can also be proved by the variation of electrical conductivity in Figure 8. It can be seen that the electrical conductivity is decreased with increasing solution temperatures. However, the electrical conductivity almost remains the same value when the solution temperature is above 1000 °C, suggesting that the alloying atoms are dissolved. The cold rolling and aging process are consequently conducted on the solution treated specimens without hot deformation, due to the excellent homogeneity.

### 4.2. The Influence of Addition of Cr on Precipitation Process

As mentioned above, the addition of a small amount of Cr to Cu-Ni-Si system alloys produces an intermetallic compound of Cr_3_Si, which is formed mainly in the liquid state during the solidification process [38]. The dispersed Cr_3_Si particles can impede grain growth and enhance the elongation of the alloy. Application of two-step aging to the Cr-added alloy increases the strength without reducing elongation by effectively decreasing the inter-precipitate spacing [11].

**Figure 9:**
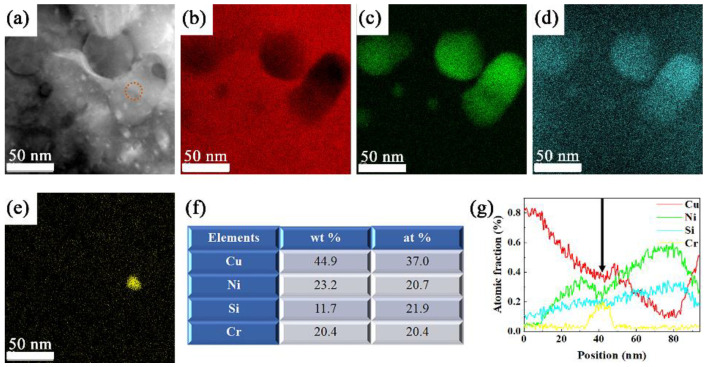
The TEM photographs showing the Ni-Si enriched precipitates in (**a**), the corresponding elements distribution of (**b**) Cu, (**c**) Ni, (**d**) Si and (**e**) Cr, and the EDS data (**f**) of the precipitate marked by orange circle in (**a**), and (**g**) linear distribution of elements as illustrated in black arrowhead in (**a**).

However, the formation sequence of Cr_3_Si and δ-Ni_2_Si is barely reported, although it is important for the further understanding of the effect of the Cr addition into Cu-Ni-Si alloys. It is found that the precipitation process of δ-Ni_2_Si is influenced by the formation of Cr_3_Si, as revealed by the TEM photographs and the corresponding chemical composition distributions of the specimen that has experienced the two steps of aging process in Figure 9. Several δ-Ni_2_Si precipitates with size from ~10 nm to ~50 nm are observed in Figure 9a. The corresponding distribution of Cu, Ni, Si, and Cr are listed in Figure 9b–e, showing a precipitate containing Cr enriched area in the central part as marked by a red dotted line circle in Figure 9a. The average chemical composition of the area marked by this red dotted line circle is listed in Figure 9f, containing ~20.4 at.% of Cr, ~20.7 at.% of Ni, ~21.9 at.% of Si, and ~37.0 at.% of Cu, suggesting coexistence of Cr_3_Si and δ-Ni_2_Si. It can be calculated that the average (Ni + Cr)/Si ≈ 1.88, which is smaller than the atomic ratio in both Ni_2_Si and Cr_3_Si. Then it is reasonable to believe that the precipitate shown in Figure 9a contains Ni_2_Si, Cr_3_Si and (Ni, Cu)_2_Si that has been reported in [6]. The linear distribution of Cu, Ni, Si, and Cr along the profile of this area, as marked by the black arrow in Figure 9a, is shown in Figure 9g. It is found that the central part with size of 8–10 nm, i.e., the position pointed by a black arrow in Figure 9g, contains about ~18 at.% of Cr, ~22 at.% of Ni, and ~20 at.% of Si (the rest is Cu). Consequently, it is reasonable to believe that, this area consists of Cr_3_Si, δ-Ni_2_Si, and a small amount of δ-(Ni, Cu)_2_Si [6]. It can be deduced from this chemical composition distribution across the precipitation that, a Cr_3_Si with size of ~5–10 nm shall be formed first during the aging process. Then the nucleation and growth of δ-Ni_2_Si precipitates occurs around the boundaries of these Cr_3_Si cores, leading to an enhanced nucleation rate. According to the calculation and experiments that has been reported [39,40], the nucleation barrier and the critical nucleus size is reduced in the heterogeneous nucleation process, thereby the nucleation rate is accelerated. Therefore, the formation of DP is suppressed by the formation of Cr_3_Si cores before δ-Ni_2_Si, which is in good accordance with the experimental results of SEM and TEM observations. The precipitation process mainly containing CP is realized in the Cu-Ni-Si alloys containing (Ni + Si) ≥ 5 wt.% herein. It is possible that larger amount of nanometer precipitates can be obtained by introducing the two steps of aging process, in order to obtain an excellent combination of mechanical properties and electrical conductivity. This study provides a promising direction for the design and optimization of Cu-Ni-Si alloys based on the further understanding of the effect of the addition of Cr.

### 4.3. The Relationship between Precipitation and Strengthening

Strengthening mechanisms in polycrystalline metallic materials usually consist of four categories: solid-solution hardening, grain-boundary hardening, dislocation hardening, and precipitation hardening. The solid-solution hardening is determined by the atomic concentration of residual elements (Ni or Si) in the matrix. The grain boundary strengthening can be described well by the classical Hall- Petch equation [27], which is determined by the grain size of the alloy. The dislocation hardening is mainly determined by the dislocation density, which is related to the deformation process and the crystallization process during aging treatment. As mentioned by several works, the contribution of the three strengthening mechanisms (i.e., solid-solution hardening, grain-boundary hardening and dislocation hardening) are calculated to be about 80–100 MPa in total, which is much lower than the strength estimated from the hardness of the alloy. Therefore, the precipitation hardening shall be the leading mechanism. It has been reported by many works [15,17,18,22,23] that the precipitation hardening mechanism of Cu-Ni-Si alloy is mainly controlled by the Orowan dislocation bypass mechanism. The shearing mechanism occurs for coherent precipitates with small radii, while the Orowan bypass mechanism operates when the coherent precipitate radius exceeds a critical value or when the precipitate is incoherent. Theoretically, it is difficult for the dislocations travelling through these Ni_2_Si precipitates by cutting them along specific crystal planes, since the Ni_2_Si precipitates show very low crystallographic symmetry.

Then the high hardening of this Cu-Ni-Si alloy can be explained according to the Orowan mechanism, which can be described as:

(1) $$\sigma \;=\frac{0.4MGb}{\pi \lambda }\frac{\ln\left(2\bar{r}/b\right)}{\sqrt{1-\nu }}$$

where *M* is the mean orientation factor (3.06 for the fcc polycrystalline matrix), *G* is the shear modulus of the matrix (44.0 GPa for Cu), *b* is the magnitude of the burgers vector (0.255 nm), and *υ* is the Poisson’s ratio of the matrix (0.33). $\bar{r}$ is the mean precipitate radius, and *λ* is the edge-to-edge inter-precipitate spacing.

**Figure 10:**
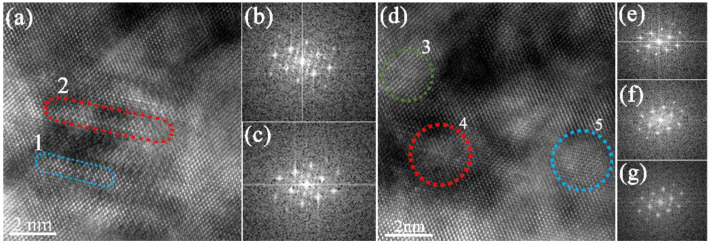
The high resolution TEM photographs of the refined Ni_2_Si precipitates in the Cu-Ni-Si alloy during the second step of aging at 450 °C for (**a**) 0.25 h, with (**b**,**c**) the corresponding SADP obtained by FFT transition of position 1 and 2, respectively, and (**d**) for 0.5 h with (**e**–**g**) the corresponding SADP obtained by FFT transition of position 3, 4 and 5, respectively.

Therefore, it is believed according to Equation (1) that, the strengthening effect is mainly affected by the mean precipitate radius and inter-precipitate spacing. It is found in our work that a large amount of refined Ni_2_Si precipitates is formed during the aging process. As shown in Figure 10a,d, refined Ni_2_Si precipitates with size of 2–5 nm in length containing 8–20 layers of atoms are observed in the specimen that has experienced two steps of aging process. The OR are shown by the SADPs in Figure 10b,c,e–g corresponding to the 1–5 positions as marked in Figure 10a,d, which is the same to the typical OR of Ni_2_Si precipitates as seen in Figure 5. The spacing between these refined precipitates are determined very small in range of 4–10 nm, as illustrated in Figure 10a,d. In this case, the refinement of these Ni_2_Si precipitates is probably due to the increase of the amount of alloying elements, since the DP is suppressed by the addition of Cr. Meanwhile, the nucleation rate is also enhanced by the formation of Cr cores, as observed in Figure 9. Then it is reasonable to believe that the precipitation hardening effect is increased since the average radius $\bar{r}$ and the edge-to-edge inter-precipitate spacing *λ* is reduced, which is in good agreement with the enhanced mechanical properties and electrical conductivity determined herein that is improved compared with some previous works.

## 5. Conclusions

The inhibition behavior on DP by the addition of Cr on the Cu-Ni-Si alloy that is prepared by HCCM continuous casting and two steps of aging treatment, is studied in this paper, in order to obtain the optimization of precipitation process and the improvement of properties. The evolution of the microstructures and precipitations, as well as the corresponding variation of mechanical properties and electrical conductivities, are studied and discussed. The following conclusions can be drawn:

(1) Both the size and amount of these δ-Ni_2_Si precipitates are smaller than those in the traditional casting ingots, due to the quite rapid cooling rate of the HCCM continuous casting method. The cold rolling and aging process are consequently conducted on the solution treated specimens without hot deformation, since the excellent homogeneity of matrix is obtained.
(2) Excellent combination of mechanical property (hardness HV 250–270) and electrical conductivity (46–47% IACS) is obtained by the first step aging at 500 °C for 0.25 h and the second step aging at 450 °C for 1 h.
(3) The nucleation and growth of δ-Ni_2_Si precipitates occurs around the boundaries of these Cr_3_Si cores, leading to an enhanced nucleation rate. The formation of DP is suppressed by the formation of Cr_3_Si cores before δ-Ni_2_Si. The precipitation process mainly containing CP is realized in the Cu-Ni-Si alloys containing (Ni + Si) ≥ 5 wt.% herein. A larger amount of nanometer precipitates can be obtained by introducing the two steps of aging process.

## Data Availability

Not applicable.
